# Origins, Trends and Perspectives of Historical-Epistemological Research on Piaget

**DOI:** 10.1007/s12124-023-09796-7

**Published:** 2023-07-27

**Authors:** Marc J. Ratcliff

**Affiliations:** Jean Piaget Center, FPSE – University of Geneva, Geneva, Switzerland

**Keywords:** Jean Piaget; research directions, International Piagetian movement, Piagetian historiography, Archives Jean Piaget, Digital Edition of Piaget’s Works

## Abstract

In this paper, several points of view are adopted to present the research directions of contemporary Piagetian historiography. This article first notes the immensity of the secondary literature (20’000 texts) related to Piaget’s work, between empirical and historical-epistemological works. In order to get a more precise idea, we carried out various analyses of the scope of influence of his work: on the 2000 keywords of the thesaurus of secondary literature from 1945 to 2012; on the reception of Piaget’s idea in France between 1920 and 1940; and on the disciplines of insertion of a thousand works from his private library, which were dedicated to him. These analyses corroborate Piaget’s multidisciplinary impact in the 20th century science and culture and indicate the existence of an international Piagetian movement, which has not yet been studied. The factors of production and the recent orientations of the secondary literature are then discussed, showing the changes in the historiography of the last 50 years. Finally, future research directions are outlined between the exploitation of unknown archives and the use of a digital edition of Piaget’s work, which is currently underway.

## An Abundant Historiography

The first observation that can be made about Piagetian historiography is that much has been written and a large number of themes have been addressed. There are intellectual biographies (Barrelet & Perret-Clermont, 1996; Ducret, 1990; Kohler, 2008; Müller et al., 2009; Vidal, 1994), numerous reception studies (Burman, 2016; de Freita Campos, Lourenço & Ratcliff Accepted; Hsue, 2009; Jaccard, 2020; Parrat-Dayan, 1993; Van der Veer, 1996; Vasconcelos, 1996), works oriented to the institutional dimension, including the Institut Rousseau, the International Center for Epistemology and the International Bureau of Education (Burman, 2021; Hofstetter & Erhise, 2022; Hofstetter et al., 2012; Ratcliff & Tau, 2018), works on disciples of Piaget (Heinzmann, 2013; Hof, 2020; Latala, 2018; Ratcliff and Tau, 2021; Ratcliff, 2010; Tryphon and Vonèche, 2000), on its relationship to psychoanalysis (Amann-Gainotti, 1998; Schepeler, 1993; Vidal, 1995), on its relationship to the media (Noël, 2020), on the Piagetian method (Bond & Tryphon, 2009; Mayer, 2005; Morelli, 2018; Vollmers, 1992) or education (Parrat, 1998; Xypas, 2001). There are countless publications on Piagetian issues, for example the conception of the social (Joly & Lebaron, 2021; Kitchener, 2009; Moessinger, 2021) discussed since the 1970s; on epistemology (Ducret, 2011; Duveen, 2000; Ratcliff, 2006; Richelle, 2000; Smith, 1993), on biology (Burman, 2013; Messerly, 2009), as well as on Piagetian concepts such as egocentrism, equilibration, play, symbolic function, adaptation theory, cooperation, not to mention cognitive domains such as causality, time, quantities, space, etc.

## A Historical-Epistemological Historiography

In this historiography, part of the secondary literature crosses a historical methodology of identification and cross-checking of facts, with an epistemological reflection. As a result, these works are stretched between two poles of an axis where the historical tendency – history of ideas, intellectual, institutional or social history – is opposed to the epistemological tendency. The latter is concerned with thinking about concepts, modeling, and systematization of the object without paying much attention to contexts and history, while, on the other hand, the historical trend is concerned with determining facts and their development in relation to their context without worrying too much about epistemological issues. These two dimensions takes various proportions, although there are works with a solely epistemological or solely historical valence. But generally speaking, Piagetian historiography is *historical–epistemological*, and extends between an epistemological and a historical pole.

A second characteristic of the secondary literature is that, while scientific works, notably of psychology of all tendencies, emerged from the 1920s and took off during the 1960s, historical-epistemological works appeared during the 1970s, between interviews of Piaget and works on the Piagetian approach and concepts. They increased significantly in the last two decades of the 20^e^ century. However, this body of work represents only a small part of the entire secondary literature on Piaget.

## The Piaget Archives Catalog, an Image of the Interdisciplinary Scope

Faced with this situation, i.e. with the flood of publications appearing in numerous journals, the creation, during Piaget’s lifetime, of the Archives Jean Piaget (AJP) in 1974 by Bärbel Inhelder and Olivier Reverdin was the sign of a centralization process of all these data. The AJP took on the mission of creating a research tool for the secondary literature, at a time when historiography was emergeing. This tool was named the *Catalogue of the Archives Piaget* and has been systematically maintained to this day. It has been published annually, listing all secondary literature citing Piaget. It was published in paper format from 1974 to 2006 and its records were then integrated into the *Alexandrie* database of the AJP.^1^ The interest of this repertory lies in its indexing by keywords, the texts were assiduously read by the Archives’ team. Covering a period from 1945 to 2020, and used reference and/or quotation of Piaget as an inclusion criterion. The number of texts – articles, chapters or monographs – collected and indexed is about 20’000 and the thesaurus contains 2000 keywords.

**Fig. 1:**
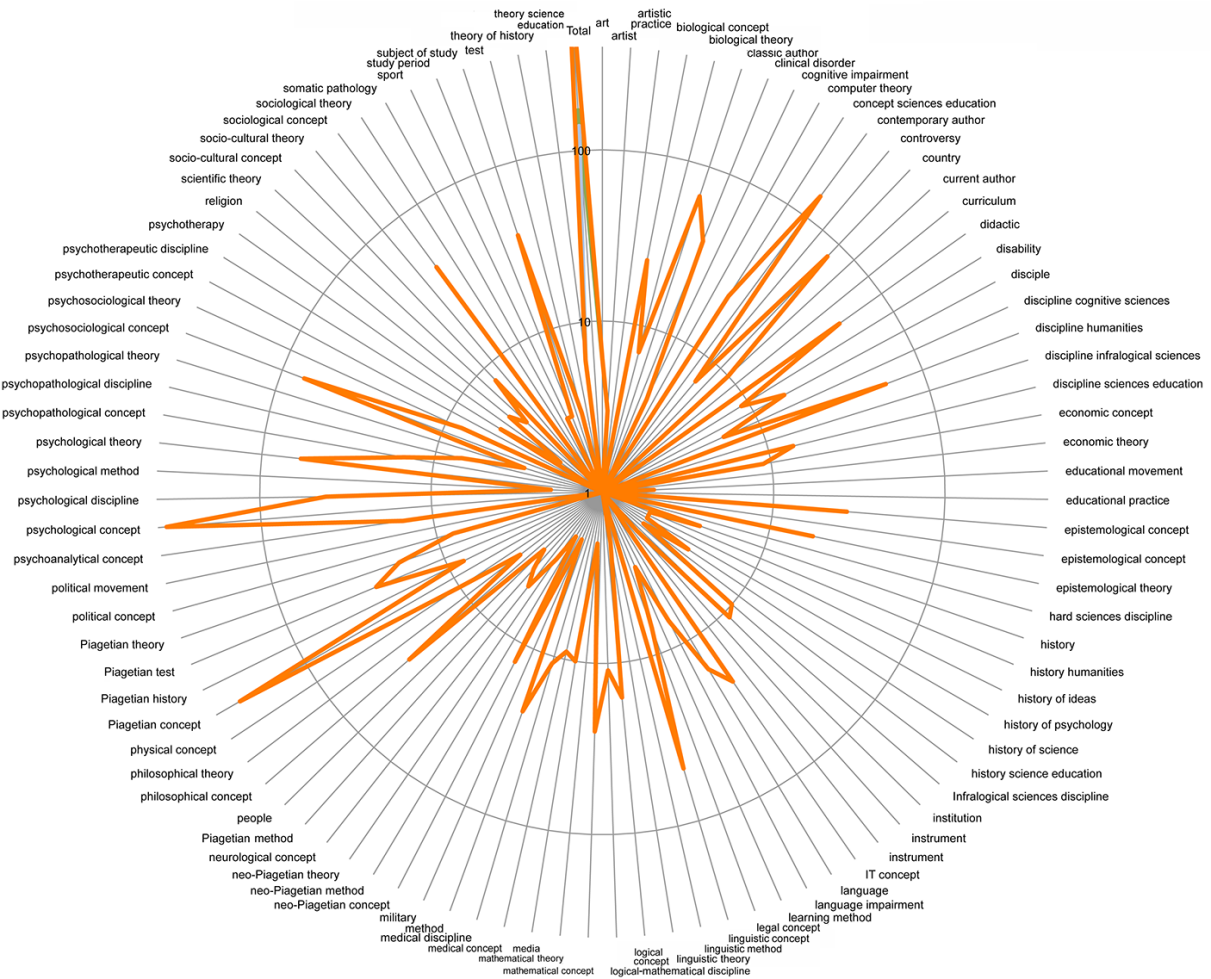
Thematic variability of the thesaurus covering the keywords of the AJP catalog for the period 1945–2012, logarithmic scale

A good part of the secondary literature listed in the catalog concerns scientific works related to Piaget and, not surprisingly, psychology, but not exclusively. Often adopting the form of an article, these are empirical researches working on a Piagetian problem or inspired by a Piagetian problematic. They attest to the extent of Piagetian influence on psychology – as well as on various scientific disciplines. Yet, the data listed and indexed have a double, scientific and historical value, because they attest to the great variability of disciplines making the Piagetian movement. This thematic variability, covering more than 70 years of secondary literature, can be measured by a typology of the thesaurus (Fig. 1).

The keywords have been organized into themes (labeled around the circle). *Psychological concepts* show the highest frequency, followed by *Piagetian concepts* and *contemporary authors*; three themes have an equivalent frequency, *psychological theories*, *human science disciplines* and *psychosociological concepts*. The *epistemological* dimension is found scattered in many themes while the *historical* dimension – at the right of the graph – is less fed proportionally to the conceptual themes.

## The Interdisciplinary Scope

The question of the interdisciplinary scope of Piaget’s work has been addressed from studies in the history of ideas (Ducret, 1984), using digital humanities (Burman, 2016) or exploring Piaget’s interdisciplinarity (Ratcliff & Burman, 2017). Indeed, Piaget, who published several articles between 1966 and 1978 on interdisciplinarity, was himself proficient in a wide range of disciplines – malacology, biology, psychology, logic, epistemology – even if for some of them, for example logic, he was neglected by his contemporaries (Ratcliff, 2016a; Burman, 2016). To contribute to an overview of this scope in view of research directions we can rephrase this question as follows: which disciplines have been influenced by Piaget’s work? To answer this question we shall cross together three investigations, from three different methodologies.

The first answer emanates from Fig. 1, which organized in categories the keywords of all secondary literature from 1945 to 2012. This graph confirmed that, from 1945 onwards, the thematic scope remained broad, while also being subject to specializations, for example studies on method and tests, on cognitive disorders, on the relationship between Piaget and contemporary authors, on the socio-cultural movement, etc. Mirrored by the keywords, the scope is immense.

**Fig. 2:**
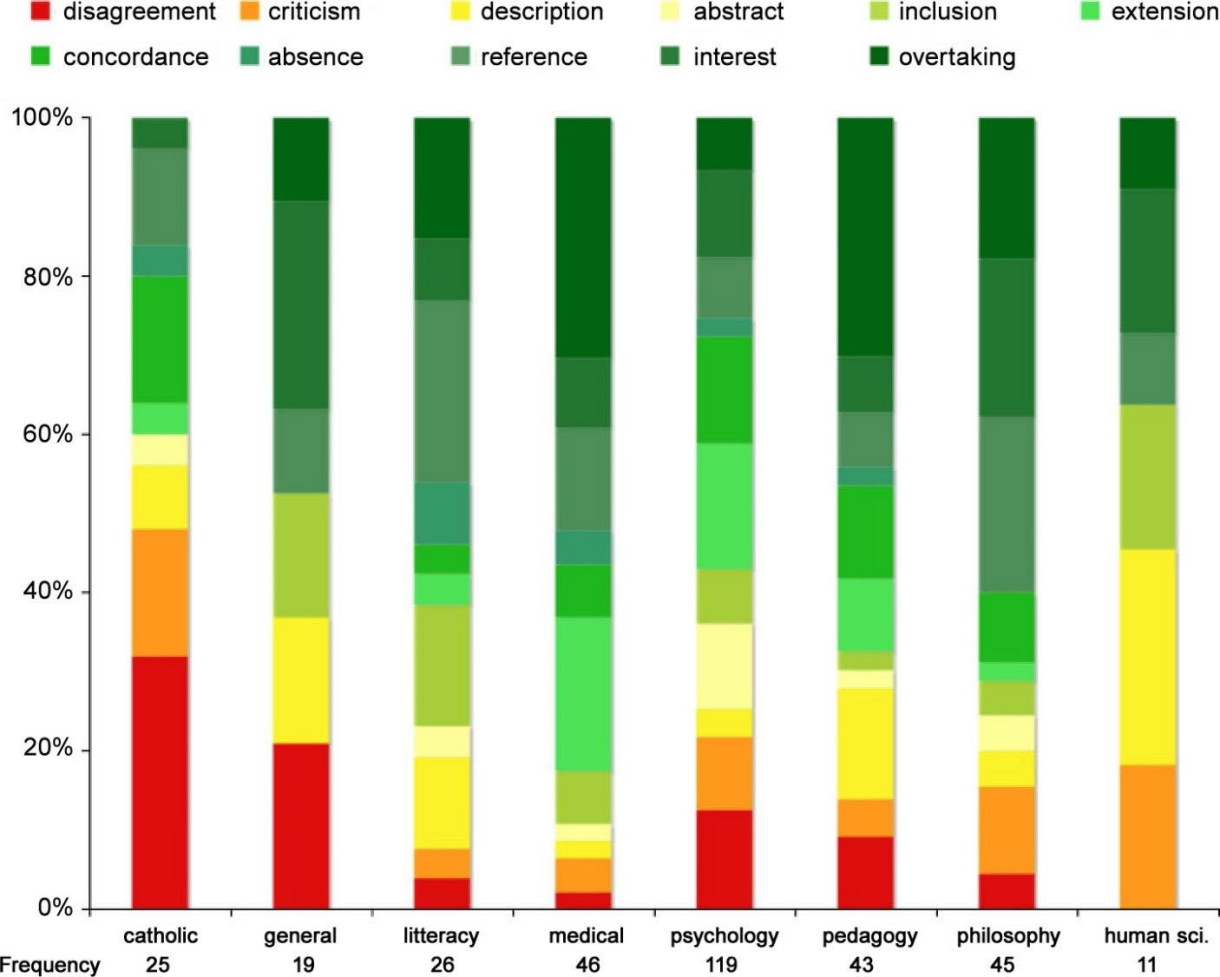
Distribution of the evaluations of Piaget’s work from 1920 to 1940 in the French environment according to the reception fields, in % and frequency

But what happened before 1945? A study on the reception of Piaget’s work during interwar in the French environment provided an answer. As the following graph (Fig. 2) shows, from 1920 onwards, the reception of Piaget’s work was also very diverse.

The reception, which was largely concentrated in psychology journals, was also important in pedagogy, philosophy and medicine (with a strong adhesion), and affected literature, the Catholic world (with the most important opposition) and the human sciences.^2^ This graph thus provided a second answer: during the interwar period, the perimeter was wide, Piaget was read by authors from very different disciplines.

**Fig. 3:**
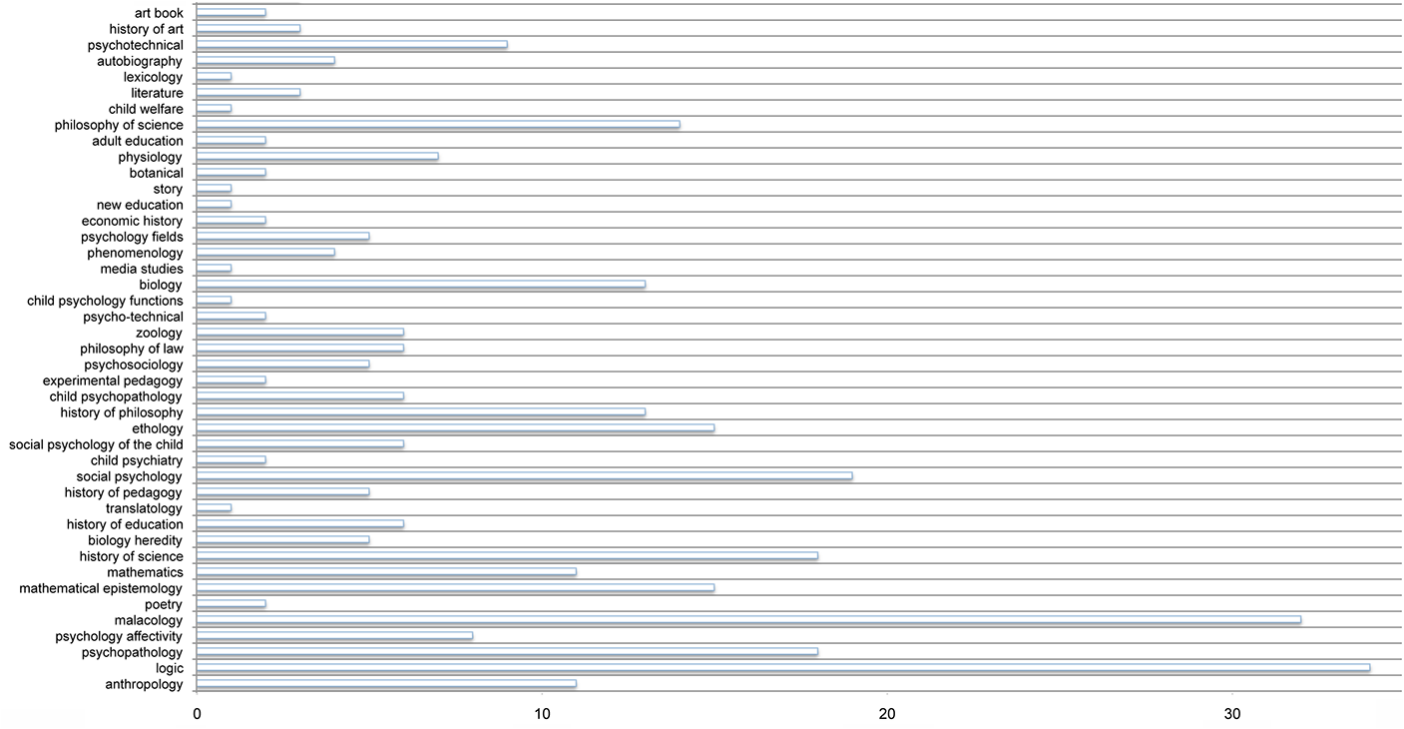
Cross-section of the categorization of works dedicated to Piaget

A final answer comes from a new series of sources: the *dedicated* books and articles from Jean Piaget’s private library, which attest to the recognition of an author, without excluding criticism. There are nearly a thousand of them, published from about 1912 to 1980. A first analysis of the disciplines in which these texts are inserted, still reserves new surprises: the dispersion allied to the degree of specialization is maximum. The graph was so big that it could not contain all the disciplines without hindering its readability. It was therefore cut up to make it readable, and showed another facet of the scope of Piagetian work, the *multiple inspiration* that authors from radically different disciplines found in it. For, far from inspiring only psychology, there appears in fact, in addition to the classic Piagetian disciplines mentioned above, a dramatic multidisciplinary dispersion: the authors came from *more than 200 disciplines* including archaeology, ethics, curriculum, creativity, ethnology, children’s literature, linguistics, management, mathematics, physics, poetics, arts, child protection, numerous sub-disciplines of theoretical or applied psychology, narrative, novel, design, science of antiquity, judaïsm, sociology of childhood, translation, etc. Here is a partial graph of this series (Fig. 3) that shows 42 out of the 203 disciplines.

## The International Piagetian Movement

The extension of the interdisciplinary scope is therefore uncommensurable to the clichés that circulate today about the status, importance and interest of Piagetian thought. In fact, people wrote about Piaget since First World War, starting with biologists, philosophers and journalists who commented on his articles and essays. In France, from the 1920s onwards, he was considered a *classic* author from 1925 onwards – it was even Wallon (1927) who wrote “Piaget’s books immediately and rightly became classics” –, and commentators and critics never ceased to confront him. From Second World War onwards, and even more so during the 1960s when Piaget was rediscovered in the United States with what is known as the “New Theory” (Beilin, 1992), the secondary *scientific* literature inspired by Piagetianism or quoting Piaget has continued to grow. As early as the 1940s, many of Piaget’s disciples, who had completed a thesis with him, published their work under the influence of the constructivist framework and applying it to very diverse domains, between clinical, perception, didactics, mathematics, psycholinguistic or microgenetics (Inhelder & Cellerier, 1992): the number of publications always increased. From 1971 onwards, after the completion of Piaget’s research program in the early sixties on perceptual mechanisms (Ratcliff & Hauert, 2006) and a period of transition, the journal *Archives de psychologie* became the voice of the Piagetians in the 1970s, after having been the organ of the Geneva psychology laboratory since 1941. All this points to the existence of an *international Piagetian movement* in the 20th century, the history of which has never been made, masked by clichés such as ‘stage theory’ and drowned out by the *normalization of constructivism* taken up in many disciplines of the human sciences. Of the secondary literature – 20’000 references in the 20th century – many papers reflect this Piagetian movement.

### The Factors of Production of Secondary Literature

The large production of secondary literature has multifactorial causes, among which:

- The mass and constancy of Piaget’s work, which over nearly 70 years, has resulted in the publication of nearly 80 works written alone, in collaboration or collectively, and 500 articles. They are listed in the *Piaget Bibliography* (1989) which has served as a reference for all the websites that have followed.
- Often at the forefront of current events, the contents of Piagetian texts have fascinated and *inspired* generations of psychologists, pedagogues, scientists, philosophers and others, men and women of all disciplines. Appealing were: the constructivist theory marked by the continuity between scientific and non-scientific thought, by the sense of structure, process and genesis, questioning the foundations of science and the categories of thought, and forming a dynamic tertium between the traditional postures of empiricism and nativism; the theoretical coherence and rigor in the treatment of issues, conceptual, experimental or formal; the question of the epistemic community thematized under the name of *epistemic subject*; the pedagogical viewpoint which, participating to the new education trend, saw the human being as a social and cooperative actor of his destiny, in a humanist vision defending democratic and egalitarian values.
- The role of the disciples and of the research culture. Piaget’s work involved 350 direct collaborators, male and female, whose role was growing until the end of the 20th century, extended by the creation of institutes and societies such as the *Jean Piaget Society*. In 2023, a *Jean Piaget Research Center* was created at the East China University. The role of the disciples is inseparable from that of the internal dynamics of the Piagetian means of production, which established at the Rousseau Institute an attractive research culture that forms the second school of Geneva, with its multiple traits: simplicity, sociability, charisma, Piagetian spirit, a system of adaptive methods, and a balance of emic (listening) and etic (rigorous objectivity) approaches.
- The multiplicity of translations is a sign of the Piagetian movement. They began in the 1920s, thanks to the training of Piaget’s students at the Rousseau Institute who translated his texts back home. Now done in about thirty languages, the translation process has fed the international production of secondary literature and continues to be fed today, including misunderstandings related to the quality of the translations (Smith, 2009). Another obstacle related to languages is the existence of national historiographies which, through an effect of *historiographic differential* (Ratcliff, 2016b), frequently ignore other national historiographies, according to mechanisms partly linked to the hegemony of English.

### Recent Orientations

Since the 2000s, there has been a certain loss of momentum in empirical work on Piagetian concepts. The origin of this is multiple, between an outdatedness of Piaget’s work which stereotyped it, a competition with other research trends (socio-cultural, neuropsychological, embodied cognition and mainstream), a change of academic culture where the reading unit of psychologists has become the article and the abstract and no longer the book, a certain loss of topicality. But if there is a loss of momentum in *empirical* work, since 2010 the same cannot be said of historical-epistemological work, that shows a certain vitality. First of all, there is a new interest in the actors and actresses around Piaget (Evert Beth, Alina Szeminska, Jean-Blaise Grize, Seymour Papert, Vinh Bang, Marc Lambercier, etc.) other than the one for Inhelder already explored by Tryphon, Vonèche and Bond; in the field of educational sciences, Piaget is unavoidable notably for his place within educational internationalism (Hofstetter, Droux & Christian, 2020); recent theses on Piaget had strong historical-epistemological value (Burman, 2016; Dell’Omodarme, 2014; Morelli, 2018; Noël, 2020); Piaget’s influence on many countries is illustrated by works on his reception; there are editorial undertakings, such as the translation of two-thirds of Piaget’s work into Mandarin (Ratcliff, 2020). And finally, between 2012 and 2016 the AJP received a vast archive of Piaget and his relatives: the Piaget Family Donation, that fundamentally modified the representations of Piaget’s character and work, and opened new perspectives. Our own research directions are oriented towards the black box of Piaget’s personality and mode of interaction reconstructed with a microhistorical methodology within a research culture – the set of means of producing facts – that he established in Geneva during the 20th century. What emerged is a profoundly humanistic character, with a horizontal relational power and an ability to improve the other, which we have named the *Maurice Effect* after one of his assistants told me that “Piaget made you clever” (Ratcliff, 2022). The Piagetian research culture constitutes in itself an original model of research organization, strongly heuristic, which supposes, in a constructivist view, a different relationship to the childhood continent, balancing etic and emic approaches, than that of mainstream research. More generally, by making it possible to explore new issues, the donation contributed to a change of era in the historioraphy.

### Perspectives: Changing Environments for Piagetian Historiography

The contexts of Piagetian historiography have changed significantly over the course of their history. They have been affected both by changes in the historiographic and material environment and by archival discoveries. Four periods can be distinguished.

1. Until the 1980s, the historiographical environment was the paper culture, handwritten and typed writing. The very sources of historiography were mainly the published works of Piaget – his autobiographical writings (Piaget, 1966) – the works of the authors he quoted and secondary literature, and some elements of oral history.
2. During the 1990s, in view of the centenary of Piaget’s birth (1996), the historiography also unfolded in line with the development of computer culture. One began to benefit from archival sources discovered in archival repositories external to the main repository, the Piaget Archives, which at that time contained only a few manuscripts and letters.
3. The year 2012 marked an important turning point with the signing of an agreement between the AJP and the Piaget family (Jacqueline Cérésole-Piaget and Lucienne Piaget) stating a donation of the entire archive contained in Jean Piaget’s house in Pinchat. Nearly 80’000 documents, including Piaget’s library, thousands of letters and hundreds of manuscripts were gradually being processed. At the same time, the development of computer culture and the Internet, the standardization of the PDF format and its availability on the website of the *Fondation Jean Piaget pour recherche psychologique et épistémologique*, thanks to the work of Jean-Jacques Ducret, provided much greater access to many of Piaget’s texts and works. New external archives – for example the International Bureau of Education directed by Piaget for 40 years – and internal archives – the Piaget family donation – were beginning to be exploited.
4. As a future perspective, the next step is linked to the ENOP project, the *Digital Edition of Piaget’s Works*. With this project underway, supported by the University of Geneva as well as by academic and private foundations in Geneva, in 2025 the Jean Piaget Center and Archives will make available to the public on its website the online edition of Piaget’s complete works, in TEI format, which is the standard format for digital humanities. The site will be equipped with a textometry laboratory allowing to analyze the whole work, linked with the texts of the secondary literature and the archives. This will be a major contribution to the Digital University of the 21th century, which will open up infinite possibilities for exploitation by providing new directions for research.

## Conclusion

With respect to the question of research directions, this paper shows several avenues. First of all, the field is not virgin. An immense amount of Piagetian themes have been tackled and a lot of historical-epistemological work has been done since the 1970s, often divided into national historiographies that have little contact with each other. However, the tools for exploring the secondary literature suffer from a lack of visibility that will be remedied with the ENOP project.

In qualitative terms, the large interdisciplinary scope has the potential to enriching historical-epistemological research with new orientations, thus taking it out of the closed circle of mainstream or socio-cultural psychology. There is an immense research territory there, waiting to be explored, including the avenues of the Piagetian multiple inspiration.

In historical terms, the explanatory factors responsible for the production of secondary literature needs to be investigated in greater depth. In particular, the notion of the *international Piagetian movement* requires historical investigations and studies at the local, national and international levels, taking into account material and symbolic circulations. A number of works exist, notably for the Western world and South America, but we know few things about Piaget in the Asian countries where their access to Piagetian thought was late – except for Japan who started during the fifties – and deserves investigation.

In terms of perspectives, the Centre Jean Piaget of the University of Geneva, which includes the Jean Piaget Archives as a historical pole, will be called to an increased presence with the ENOP project. This project has a vocation of unification, of Piaget’s texts, of secondary literature and of the archives. With its vocation of encouraging research, both historical and psychological, it will provide access to reliable sources, thus making it possible to support a number of arguments in many debates – whether it be the role of constructivism in early skills, the role of the social or the neurological in development, or the relationship between language and thought – as well as pertinent historical facts. The ENOP project with its textometry laboratory will allow to answer relevant questions oriented by previous historiography as well as to explore new questions resulting from bottom-up research. And perhaps it is a quote from Piaget (1928: 202) comparing the effects of constraint and cooperation that best illustrates its potential: “The commitments I make to constraint may be painful but I know where they lead me. The commitments I make through cooperation lead me I don’t know where”.

## Footnotes

1 https://ael.archivespiaget.ch/dyn/login.seam?cid=84.

2 Ratcliff, Accepted. The colors, from red to green, report the degrees of endorsement of Piagetian work by authors of secondary literature (reviews and articles). The ‘absence’ category refers to the fact that an author mentionned that Piaget *should* have been cited by a third author.
